# Certain Soil Surfactants Could Become a Source of Soil Water Repellency after Repeated Application

**DOI:** 10.3390/nano11102577

**Published:** 2021-09-30

**Authors:** Enzhan Song, Keith W. Goyne, Robert J. Kremer, Stephen H. Anderson, Xi Xiong

**Affiliations:** 1 Agriculture Development Group Inc., Eltopia, WA 99330, USA; songe@centurytel.net; 2 Department of Forest Resources and Environmental Conservation, Virginia Polytechnic Institute and State University, Blacksburg, VA 24061, USA; goynek@vt.edu; 3 School of Natural Resources, University of Missouri, Columbia, MO 65211, USA; KremerR@missouri.edu (R.J.K.); AndersonS@missouri.edu (S.H.A.); 4 Division of Plant Sciences & Technology, University of Missouri, Columbia, MO 65211, USA

**Keywords:** critical micelle concentration, hydrophobic organic coating, repeated wetting agent application, soil water repellency, surfactant sorption

## Abstract

Repeated application of soil surfactants, or wetting agents, is a common practice for alleviating soil water repellency associated with soil organic coatings. However, wetting agents are organic compounds that may also coat soil particle surfaces and reduce wettability. For this experiment, hydrophobic sands from the field and fresh, wettable sands were collected and treated with either a polyoxyalkylene polymer (PoAP) or alkyl block polymer (ABP) wetting agent, or water only treatments served as a control. Following repeated treatment application and sequential washings, dissolved and particulate organic carbon (OC) were detected in the leachates of both sand systems. The total amount of OC recovered in leachates was 88% or less than the OC introduced by the wetting agents, indicating sorption of wetting agent monomers to soil particle surfaces regardless of soil hydrophobicity status. While ABP treatment did not alter solid phase organic carbon (SOC) in the sands studied, PoAP application increased SOC by 16% and 45% which was visible in scanning electronic microscopy images, for hydrophobic and wettable sands, respectively. PoAP application also increased the hydrophobicity of both sands that were studied. In contrast, ABP treatment increased the wettability of hydrophobic sand. Our results provide strong evidence that certain wetting agents may increase soil hydrophobicity and exacerbate wettability challenges if used repeatedly over time.

## 1. Introduction

Soil water repellency (SWR) is a common issue for agricultural lands, pastures, forests, and other landscaped areas such as residential lawns and golf courses [1,2,3,4,5]. This condition affects soils in over 50 countries [6], but it is especially problematic in sandy soils due to the low specific surface area of sand particles [7]. Upon development of this condition, SWR typically leads to localized dry spots that are treated with wetting agents, a type of soil surfactant, to manage drought symptoms [8,9].

The formation of SWR is believed to be associated with soil microbial activities which slowly decompose plant materials and convert them to soil hydrophobic organic compounds [10,11,12,13]. Hydrophobic organic compounds include humic substances [14] and biomolecules derived from microbes and plants such as waxes [15] and fatty acids [16,17]. As components of soil organic matter, hydrophobic organic compounds tend to be nonpolar and low in solid surface free energy or, in other words, low surface tension [1]. Over time, these organic compounds coat the surfaces of mineral particles, resulting in a decrease of surface tension and an increase of overall soil hydrophobicity [18].

Most wetting agents are formulated with non-ionic surfactants consisting of amphiphilic molecules with polar hydrophilic heads and non-polar lipophilic/hydrophobic tails. Such characteristics allow wetting agents to attach their lipophilic tails onto non-polar hydrophobic particle surfaces while their polar ends interact with soil water [19] to improve wetting [6,8]. Another group of specialized wetting agents, such as polyoxyalkylene polymer (PoAP), also aims to remove hydrophobic organic compounds through solubilization/desorption, and presumably flush the organic coatings out of the system. It is, however, important to note that the amphiphilic properties of the surfactants may also increase the affinity of hydrophobic organic compounds to soil particles, thus reversing the anticipated wetting. Song et al. [20] reported that one application of PoAP reduced the SWR to a minimum level, however, at a cost of 27% increase in solid phase organic carbon (SOC) in the treated sands, suggesting a strong sorption of PoAP molecules to hydrophobic sand surfaces. This counteraction is believed to occur more often following repeated applications of wetting agents, or wetting agents containing surfactants that strongly sorb to surfaces [21].

Previous research investigating the sorption of non-ionic surfactants to different soil minerals found a positive correlation between the mass of mineral silica and the sorption of nonylphenol ethoxylates that are commonly used in wetting agents [22]. Considering that wetting agents are primarily applied to silica sand-based soil media, the adsorption of surfactant molecules onto sand surfaces is expected. Furthermore, Sun et al. [23] found a positive correlation between soil–water distribution coefficients and surfactant concentration when a micelle-forming surfactant was applied below its critical micelle concentration (CMC). The CMC is the minimum aqueous concentration of a surfactant when monomers start to saturate in the solution and aggregate into micelles [24,25]. The CMC value is determined by surfactant structure and composition and is influenced by environmental conditions such as temperature and solution ionic strength [24,25]. The micelles orient in a way where monomer non-polar tails are in maximum contact with each other creating a hydrocarbon-like core while their hydrophilic polar heads face outside and interact with water [19]. However, when the same surfactant was applied above the CMC, a negative correlation between soil–water distribution coefficients and surfactant concentration was observed, indicating the possible solubilization/partitioning of hydrophobic organic compounds and potential precipitation onto the soil particles. Under field conditions, wetting agents are often repeatedly applied, which increases the chance of accumulation of wetting agent molecules above their CMC in the system. Therefore, the objectives of this study were to investigate the potential sorption effect of selected wetting agents after repeated application, and to discover their influences on hydrophobic organic coatings on sand surfaces and hence wettability of the sands.

## 2. Materials and Methods

Hydrophobic sands were obtained by homogenizing and drying sands collected from a water repellent area on a sand-based putting green constructed to meet the United States Golf Association specifications. The consistent and strong hydrophobicity level of the sands was validated via the molarity of ethanol droplet test (MED) with a value of 2.2 molar (M) [26,27].

To simulate water movement as influenced by wetting agents, 254 g of the prepared hydrophobic sand or fresh, wettable sand that was obtained from the same source as the hydrophobic sand, were packed into PVC tubes (Georg Fischer Harvel LLC, Easton, PA, USA) to a depth of 7.80 cm or 11.20 cm, respectively. The tubes had 5.08 cm internal diameter and 0.48 cm thickness, and one end was covered by a layer of fabric with maximum openings < 0.05 mm to retain the sands. Bulk densities of hydrophobic and fresh sand columns were 1.66 g cm^−3^ or 1.74 g cm^−3^, and particle densities of hydrophobic and fresh sand were measured as 2.68 g cm^−3^ or 2.76 g cm^−3^, respectively. Subsequently, the porosity was determined as 38% for hydrophobic sand columns and 37% for fresh sand columns, which were equivalent to a pore volume of 58 and 54 mL for hydrophobic and fresh sand columns, respectively (Table 1).

Surfactants applied included compounds in the chemical groups of alkyl block polymer (ABP) (Matador; 100% alkyl block polymer; EnP Investments LLC., Mendota, IL, USA), PoAP (OARS; 80% polyoxyalkylene polymers and 10% potassium salt of alkyl substituted maleic acid; AQUA-AID Inc., Rocky Mount, NC, USA), and a distilled, deionized water (DDW) control. Liquid surface tension of the two wetting agents at 0.5×, 1×, 2×, 3× and 4× label rates, which were equivalent to 12.5, 25.0, 50.0, 75.0, and 100.0 mL L^−1^ for ABP, and 14.4, 28.8, 57.6, 86.4, and 115.2 mL L^−1^ for PoAP, were determined by using an Attension Theta Lite tensiometer (Biolin Scientific, Inc., Linthicum Heights, MD, USA).

**Figure 1:**
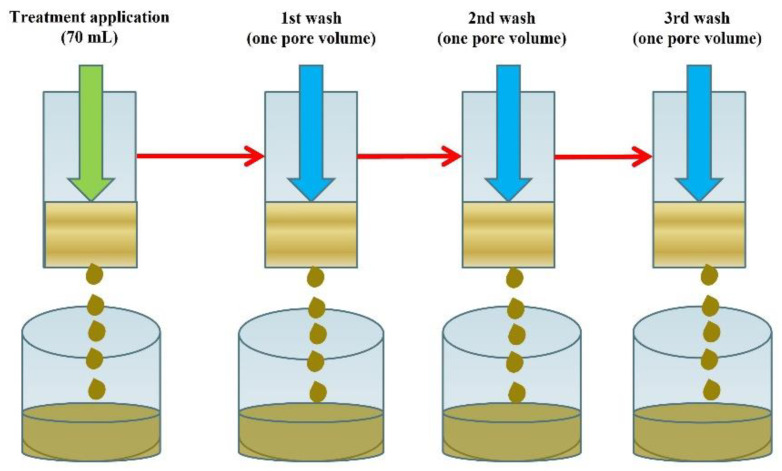
Schematic illustration of the experiment process. The sand columns were subjected to treatment application and 24 h after, deionized, distilled water at one pore volume (58 mL for hydrophobic sand and 54 mL for fresh sand) were used to wash the sand columns three times at a 30-min interval between each wash. Without disturbing the sands, the sand columns were then subjected to oven drying at 50 °C for 7 days, before additional treatment applications and triple washing occurred, as described for the first cycle. The entire process was carried out three times.

Label rate (1× rate) solutions of ABP or PoAP were prepared and 70 mL of each solution, along with DDW as a control, were applied slowly and carefully to the top of individual fresh or hydrophobic sand columns to prevent the formation of standing water. After 24 h, treated sand columns were rinsed three times using one pore volume of DDW applied the same manner described above, with a 30-min interval between each wash event. Column leachates were collected for further analysis during treatment application and the three washes [28]. Without disturbing the sands, the triple-washed sand columns were then dried at 50 °C for 7 days. After which, the sand columns were carefully re-assembled and the entire process, including treatment application and triple washing and oven drying, was repeated twice for all treatments. This was performed to simulate multiple wetting agent applications in each growing season under field conditions. Consequently, a total of three treatment applications and nine washes were carried out (Figure 1).

Leachates collected from all three treatment applications and nine washing events were immediately acidified to pH < 2 and stored at 4 °C in the dark. The leachate volumes were recorded, prior to determination of dissolved organic carbon (DOC) and particulate organic carbon (POC) content of the leachates. Measurement of POC was performed by first centrifuging the leachate at 10,000× *g* for 15 min at 4 °C, followed by removal of the supernatant. Complete separation of POC from the solution was confirmed by centrifuging 0.5 mL supernatant at 10,000× *g* using nylon membrane filter centrifuge tubes (Corning^®^ Costar^®^ Spin-X^®^ centrifuge tube filters; Corning Inc.; Glendale, AZ, USA) with a pore size of 0.45 µm. No particles were obtained on the filter after centrifugation, indicating a satisfactory extraction of POC from the leachates. Solids POC separated from the solutions were dried at 105 °C for 12 h, and weighed and combusted under 550 °C for 12 h, before being allowed to cool and then reweighed [29]. Particulate organic matter of the leachate was calculated as the mass loss after combustion, and the POC content was calculated using the conversion factor of 58% [30]. Determination of DOC was carried out by measuring the organic carbon in the 0.5 mL centrifuged and filtered leachate using a TOC-VWP analyzer (Shimadzu Corp.; Kyoto, Japan) equipped with an autosampler ASI-V. The total DOC mass of each leachate sample was then determined by multiplying by the corresponding leachate volumes.

After completing the entire experiment, sand samples were collected and solid phase organic carbon was determined by utilizing a LECO TruSpec CN Carbon/Nitrogen analyzer (LECO Corporation; St. Joseph, MI, USA). Hydrophobicity of the treated sands was determined via the MED test. As a comparison, SOC and MED of untreated sand samples were determined using the same methods described above. Additionally, sand samples were subjected to scanning electronic microscopic analysis (SEM), using a Hitachi S-4700 field emission scanning electron microscope (Hitachi America Ltd., White Plains, NY, USA) with a variety of non-cryo sample holders.

The experimental design was a complete randomized design with three replications, and the entire experiment was repeated once. Analysis of variance was conducted using the Proc GLM procedure of SAS 9.4 (SAS Institute, Cary, NC, USA). No interactions between treatment and run of experiment were found for all measured parameters, thus data from the two runs were pooled. Significant mean separations were performed based on Fisher’s Protected LSD at *p* ≤ 0.05.

## 3. Results and Discussion

### 3.1. Surface Tension-Concentration Relationship

**Figure 2:**
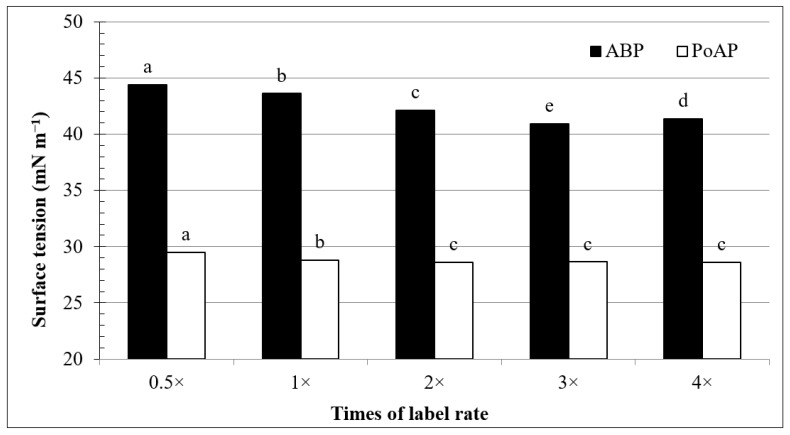
Surface tension of alkyl block polymer (ABP) and polyoxyalkylene polymer (PoAP) influenced by their concentrations at 0.5×, 1×, 2×, 3×, and 4× label suggested rates, which corresponded to 12.5, 25.0, 50.0, 75.0, and 100.0 mL L^−1^ for ABP, and 14.4, 28.8, 57.6, 86.4, and 115.2 mL L^−1^ for PoAP, respectively. Bars labeled by the same letter for each wetting agent are not significantly different based on Fisher’s Protected LSD at *p* < 0.05.

Surface tensions of ABP at 0.5× to 4× label rates ranged from 44.4 and 40.9 mN m^−1^, while PoAP for the same rates ranged between 29.5 and 28.6 mN m^−1^ (Figure 2). All surface tension values were substantially below 73.0 mN m^−1^ for DDW tested under the same conditions, suggesting that both ABP and PoAP at those rates would significantly increase water infiltration into hydrophobic surfaces [31]. As the concentrations of the wetting agents increased from 0.5× to 2× of the label rates, surface tension showed a steady decline for both compounds (Figure 2). With further increases in concentration, the surface tension of ABP reached its lowest point at the 3× label rate and rebounded slightly, by 1%, at the 4× rate. In comparison, surface tension of PoAP was relatively constant at the rate of 2× and greater. This cessation of further decreases in surface tension despite increasing concentration often indicates that the CMC has been reached, and beyond this point surfactant monomers would form micelles that might lead to undesirable effects [32,33]. Our results suggested that the concentrations of both ABP and PoAP at label rates (1× rate) did not exceed the CMC.

### 3.2. Effects on Leachate Volume

Leachate volume indirectly reflects treatment influences on soil hydrologic aspects, such as hydraulic conductivity and water holding capacity. In the hydrophobic sand system, following the first application of ABP or PoAP at 70 mL, leachate volumes were measured at 0 or 1 mL, respectively, indicating that nearly the entire 70 mL volume of wetting agent solution applied was retained (Table 2). In comparison, 53 out of the 70 mL water applied in the control treatment was retained by the hydrophobic sand columns, suggesting reduced water holding capacity of untreated sand as previously reported [20]. Collectively, these results indicated that the water holding capacity of hydrophobic sands was improved shortly after wetting agent application. Following the first wash event, 81% of the applied water was leached from the hydrophobic sand columns receiving ABP. This was 20% greater than leachate from the columns receiving water only treatment and approximately 3× the leachate amounts from PoAP-treated sand columns. This result was intriguing as it suggested that following the first washing event, PoAP-treated sands continued to hold excessive amounts of water compared to the control, while ABP-treated sands began facilitating water movement through the sand columns. The disparity, however, started to dissipate and after the second and third washing event where ≥84% of applied water was recovered in the leachates regardless of treatment.

After oven-drying and the second treatment application, 100% or 94% of the applied wetting agent solution was retained in ABP- or PoAP-treated hydrophobic sand columns, respectively, which was similar to the first treatment application (Table 2). In contrast, water did not infiltrate into the hydrophobic sand columns receiving water, only, treatment following the first application and three wash events and hence, no leachate was collected during the second and third sequential application and wash events. Early research has suggested that high temperatures under field conditions or oven-drying of moist water-repellent soil will increase SWR [15,34]. As the sand columns were oven-dried between each treatment and wash sequence, results found in this experiment support such findings.

Following the first wash event, 78% of applied water was recovered from the leachate following ABP application. This was comparable to the result found after the first run of treatment application. However, a different pattern was shown for PoAP after the first washing event, where 81% of water was recovered from the leachate, which was nearly 3× the leachate amount recorded during the washing event of the first run of treatment application. Collectively, these results suggested that water retention of ABP following multiple runs of treatment application is more predictable, while the effect of PoAP could be variable depending on the number of treatment applications. Leachate volumes following the second and third washing showed a similar pattern, where no differences were found between the two treatments.

After another oven-drying and the third treatment application, hydrophobic sand columns receiving ABP or PoAP resulted in similar leachate patterns compared to the results recorded following the second run of treatment application.

Previous studies have reported a similar disparity among soil surfactants and their effects on soil hydraulic properties. Some reports show enhanced soil water retention and increased water holding capacity compared with untreated material without improved water movement in water repellent soil [35,36]. In contrast, others found improved water infiltration/drainage or lower soil water content in treated hydrophobic soil [37,38,39,40,41]. These results suggest that wetting agents with different chemical properties may act through different mechanisms.

In contrast, leachate patterns generated from the non-water repellent, fresh sand columns were more consistent among all treatments, including the water only treatment (Table 2). Applications of ABP or PoAP to fresh sand showed negligible effects on water retention compared with the hydrophobic sand system, suggesting minimum effects on soil without hydrophobic organic coatings. Some previous reports suggest that soil surfactants may not be beneficial in healthy, hydrophilic soils [42,43], however, this was not evident in our study based on the leachate patterns presented in Table 2.

### 3.3. Effects on DOC, POC, and SOC

Measurements of DOC and POC mass content in the leachate following treatment application and each wash were provided in Supplementary Tables (Tables S1 and S2). Summarized DOC and POC by each treatment application including the sequential washes after each of three runs were provided in Table 3. Results from water treatment were not included in this analysis as only one run of water application was carried out as explained above. Compared to PoAP, leachate collected from ABP-treated sand columns consistently showed greater amounts of DOC, regardless of the status of sand hydrophobicity. With each repeated treatment application, the amount of DOC steadily increased in leachate collected from both sand systems regardless of the applied wetting agent. This pattern indicated an initial coating/sorption of surfactant monomers on the sand surfaces, but reduced surfactant reactivities following the previous treatment were likely due to increased development of surfactants coating the sand particles. The amounts of DOC detected in the leachate from fresh sand were comparable to the leachate collected from the hydrophobic sand. This was likely due to the wetting agents applied into the sand systems, which are liquid forms of organic compounds and hence, sources of organic carbon.

The amounts of POC detected in leachate collected from fresh sand were substantially lower compared to the leachate collected from hydrophobic sand (Table 3). As both wetting agents were applied as solutions, it is reasonable to assume that a substantial amount of POC detected in this experiment originated from hydrophobic organic coatings present in hydrophobic sand, evidenced by the results shown here. Further evaluation of the data revealed that PoAP-treated fresh sand columns showed greater amounts of POC in two of three application events compared with ABP; however, this trend was reversed for hydrophobic sand. Collectively, these results indicated a greater capability of ABP for removing POC from the hydrophobic sand surfaces. Earlier literature reported that the presence of fine organic matter particles ranging in size from 0.45 µm to 0.1 mm increases the SWR by increasing the solid–liquid contact angle [44,45]. The extraction method used in this experiment detected POC within sizes ranging between 0.45 µm and 0.05 mm. Therefore, our results suggest that ABP will most likely reduce the SWR of the hydrophobic sand, due to enhanced removal of particulate organic carbon from the soil as evidenced here.

To facilitate a better understanding of the data, total dissolved, particulate, and cumulative organic carbon collected from all three treatment applications and their sequential washes were summed together and were compared with total organic carbon input from the wetting agent applications (Table 4). Compared to PoAP, a greater amount of total DOC was recovered following ABP treatment regardless of the sand system. This could be attributed to the 29% greater OC introduced by ABP applications in comparison to PoAP. Compared to hydrophobic sand, total DOC recovered was significantly higher from fresh sand system regardless of wetting agent applied. However, in absence of OC input (i.e., the water only treatment), the amount of total DOC detected in fresh sand leachates was nearly 3× greater than that recovered from hydrophobic sand. This disparity between the sands could explain the differences observed between the two sand systems following wetting agent applications.

Without addition of OC, water only treatments resulted in the same amount of total POC in both fresh and hydrophobic sands (Table 4). ABP-treated fresh sand resulted in statistically similar amounts of total POC compared to water only treatment. However, PoAP applications to fresh sand resulted in 2.5× of total POC compared to water only treatment, suggesting that a substantial portion of the total POC originated from the treatment itself. Following treatment applications to hydrophobic sand, both wetting agents removed relatively greater portions of POC compared to the water only treatment, resulting in 12× and 7× POC removed by ABP and PoAP applications, respectively. This result again indicates a potentially greater effect in reducing SWR by ABP application.

Consequently, ABP applications removed greater amounts of total OC compared to PoAP, regardless of sand system (Table 4). It is intriguing to notice that PoAP recovered greater amounts of total OC in leachate from fresh sand system compared to hydrophobic sand system, similar to the water control; however, this trend was reversed for ABP, indicating again a more effective OC removal from the hydrophobic sand following ABP applications. Regardless of wetting agents or sand systems used, however, none of the wetting agents recovered the same amount of the OC in leachate compared to the amount of OC introduced by treatment application. This result indicated that the surfactant monomers were retained in the sand system through coating/sorption, even on fresh sand with a minimum or no presence of hydrophobic organic coatings. Strong adsorption of nonionic surfactant onto clean sand has been reported previously by Bera et al., [46] who conducted X-ray diffraction and Fourier transform infrared spectroscopy experiments to confirm the adsorption of nonionic surfactant on silica sand. While the X-ray diffraction results showed a high purity of silica sand, spectra of nonionic surfactant-treated sand showed a C-H bond stretching vibration shifting from 2920 cm^−1^ to 2927 cm^−1^, in addition to a vibration at 1888 cm^−1^, due to adsorption of surfactant ethoxylate groups to the sand surface. A fitted Langmuir adsorption isotherm also showed a positive correlation between surfactant concentration and adsorption to the sand particles, but increases in adsorption ceased at the CMC. Normally, following an increase of surfactant concentration, surface tension of the surfactant solution will decrease to the CMC and reach a constant value [32,33]. According to this concept, both ABP and PoAP did not reach its CMC at label rates (Figure 2).

**Figure 3:**
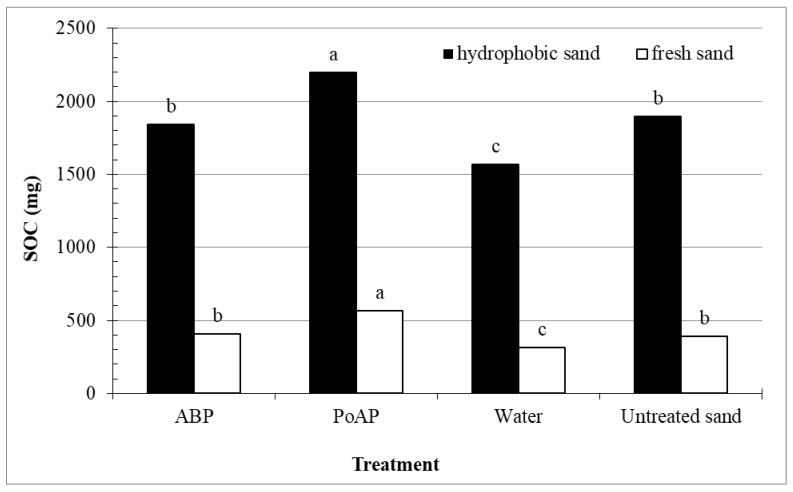
Solid phase organic carbon (SOC) of hydrophobic or fresh sand columns receiving three sequential treatment applications and following washes. Treatments included an alkyl block polymer (ABP), a polyoxyalkylene polymer (PoAP), and water, in addition to an untreated control (untreated sand). Bars labeled with the same letter for each sand were not significantly different based on Fisher’s Protected LSD at *p* < 0.05.

Detection of SOC on fresh sand revealed a low level of organic matter coating on the fresh sand surfaces (Figure 3), and water only treatment significantly reduced the SOC of the fresh sand, which supported earlier findings on POC removal (Table 4). Applications of ABP to fresh sand resulted in no SOC difference compared to untreated sands, while PoAP application increased SOC by 45%. This result again corroborates earlier findings and collectively suggest strong sorption of PoAP molecules to the sand surface, even in the minimum presence of hydrophobic organic coatings. For hydrophobic sand, in which SOC mass in the untreated hydrophobic sand was four-fold the amount in fresh sand, wetting agent applications produced a similar pattern of measured SOC among the treatments. Collectively, these results indicated a consistent sorption of PoAP molecules onto sand particle surfaces, regardless of the hydrophobicity level.

It is important to mention that despite the difficulty in separating the hydrophobic coatings that may originate from wetting agent itself, POC detected in the leachate most likely originated from hydrophobic organic coatings. As a result, we suggest a replacement effect of POC by ABP monomers that efficiently removed POC (Table 4) while maintaining the same level of SOC (Figure 3). Compared to the overall SOC quantity in the untreated hydrophobic sand system, the mass of POC that applications of ABP reduced was minimal (Table 4). However, it was reported that addition of a small concentration of POC could significantly increase SWR [11,15]. Therefore, removing even a numerically small quantity of POC by wetting agent applications could be beneficial for long-term SWR control/prevention under field conditions.

### 3.4. Effects on SWR

**Figure 4:**
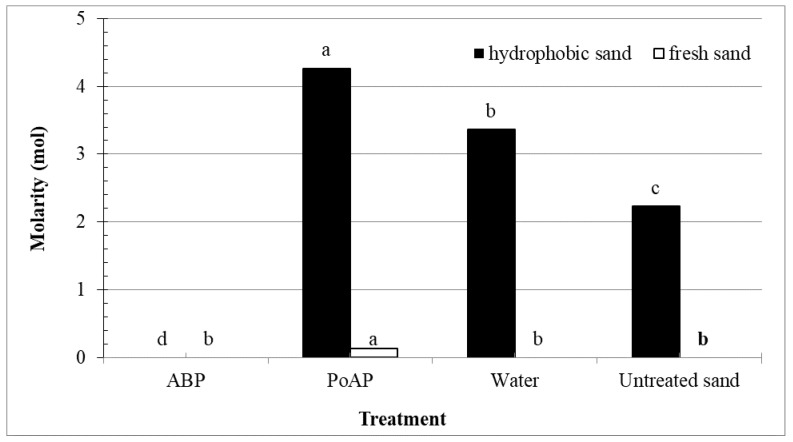
Hydrophobicity, determined by molarity of ethanol droplet (MED) test, of hydrophobic or fresh sand columns receiving three sequential treatment applications and following washes. Treatments included an alkyl block polymer (ABP), a polyoxyalkylene polymer (PoAP), and water, in addition to an untreated control (untreated sand). Bars labeled with the same letter for each sand were not significantly different based on Fisher’s Protected LSD at *p* < 0.05.

As expected, untreated fresh sand showed no water repellency, evidenced by 0 molarity based on the MED test (Figure 4). Neither water nor ABP applications changed this condition, however, PoAP applications significantly increased the MED to 0.13 M, suggesting an undesirable effect in increasing SWR even in fresh sands. Analysis of SOC showed that PoAP applications led to a numerically small increase in SOC of fresh sand (Figure 3), however, potential low accumulation of PoAP monomers on sand surfaces might lead to adverse effects on soil health in the long term. This effect is further amplified when PoAP applications were made to hydrophobic sand, which elevated the hydrophobicity of hydrophobic sand from 2.2 M for the untreated control to 4.3 M, categorized as a very severe water repellent condition defined by King [26]. In contrast, ABP-treated hydrophobic sand showed zero hydrophobicity, suggesting a desirable effect in control of SWR.

These results were very similar to those of Urrestarazu et al. [21], who discovered increased water holding capacity, reduced wettability and air capacity by reused soil surfactants, possibly due to increased water adhesion/cohesion in the micropores and even reduced pore space. Furthermore, multiple studies related to remediation of soil organic contaminants have reported little or no effect on solubilization of hydrophobic organic coatings [47], or even increased precipitation of hydrophobic organic coatings on soil particles [48] when surfactants were applied at less than the CMC. Our data suggested a stronger OC sorption effect than removal following PoAP application at less than CMC rate. More importantly, the unsaturated monomers from PoAP may act alone and/or interact with original hydrophobic organic coatings on hydrophobic sand that induced further hydrophobicity, instead of forming hydrophilic surfaces.

Even though water alone was able to remove significant amounts of OC from the hydrophobic sand system by one application and triple-wash cycle (Figure 3; Table 4), increased hydrophobicity of water-treated hydrophobic sand (Figure 4) suggests that the OC collected in the leachate of water treatment was more likely to be water extractable OC. It is important to distinguish DOC and water extractable OC in this case, as water extractable OC includes both DOC and POC that can be directly dissolved or mechanically removed by water only [49]. The limited contribution of hydrophobic substances (<30%) in water extractable OC under normal extraction temperature (20 °C, 24 h) has been reported by Nkhili et al. [50]. It was clear that the residual OC in the hydrophobic sand system after removal of water extractable OC would contribute to further induction of soil hydrophobicity.

### 3.5. Visual Images from SEM Analysis

**Figure 5:**
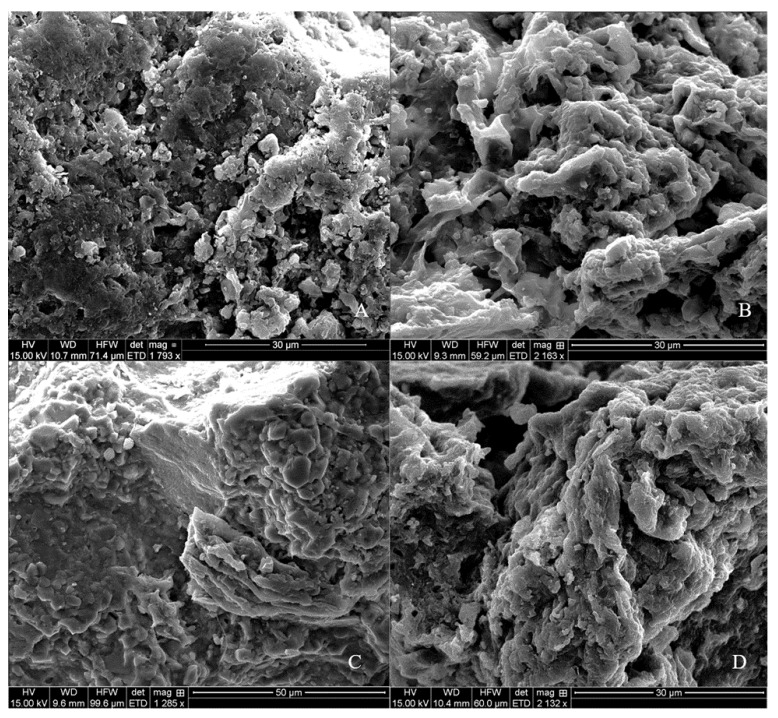
Representative scanning electronic microscopy (SEM) images of hydrophobic sand particles that received three sequential applications of wetting agent treatments and following washes. Treatments included an alkyl block polymer (ABP); (**A**), a polyoxyalkylene polymer (PoAP); (**B**), along with water (**C**) and an untreated control (**D**).

The SEM analysis showed sand particles that were enlarged >1000 times for direct observation of potential treatment effects on the organic coatings (Figure 5). The images represented ABP-treated sand surface (Figure 5A), PoAP-treated sand surface (Figure 5B), water-only treated sand surface (Figure 5C), and an untreated sand surface (Figure 5D). Changes of the surface organic coating structures showed that ABP applications resulted in more regular shapes with visible straight edges/angles which is similar to the structure of water-only treated surfaces but with significantly less continuous amorphous materials. In contrast, PoAP-treated sand appeared to have abundant amorphous structures, which is similar to the original untreated, hydrophobic sand but with smoother surfaces. It is well documented that soil organic materials have a common amorphous structure while soil minerals are usually in crystalline forms which are more regularly shaped [51,52]. These SEM images clearly showed a greater level of organic coatings on the sand surfaces after repeated PoAP applications, while repeated ABP applications resulted in a relatively cleaner sand surface. Combining the information of SEM analysis and effects on OC and SWR, it is suggested by these quantitative and qualitative data that ABP was able to remove enough detectable SWR that contribute to organic coatings on the hydrophobic sand, and PoAP was able to alter the organic coating on sand surfaces by potentially adding an unknown portion of its own organic structures into the coating system.

## 4. Conclusions

Results generated from this experiment suggest that both wetting agents, ABP and PoAP, can be retained in the sand system after repeated application. Our data further indicate that this is due to the sorption of the wetting agent monomers, which resulted in an accumulation of the wetting agent molecules on the sand surfaces, even on sand particles with minimal hydrophobicity. After repeated wetting agent application and sequential washings, organic compounds are detected in the leachate in both dissolved and particulate forms, and the latter form suggests a removal/replacement of the organic coatings from the hydrophobic sands. The two wetting agents, however, demonstrated different mechanisms. Repeated ABP application resulted in no changes in solid phase organic carbon compared to untreated sands, removed 1.7-fold of particulate organic carbon from the hydrophobic sands in comparison to PoAP, and completely reverted the hydrophobic sands to wettable medium. In contrast, repeated PoAP application elevated the amount of organic coatings on the hydrophobic sand surfaces, and this effect was clearly demonstrated in scanning electronic microscopy images. Ultimately, following repeated PoAP application, hydrophobicity of the hydrophobic sands was nearly doubled, and a measurable water repellency was established for originally wettable sands. Collectively, our results strongly suggest that certain wetting agents, such as ABP, can be safely applied repeatedly with consistent and desirable effects that alleviate soil hydrophobicity, and ultimately improve wetting. Other wetting agents such as PoAP might become a source of hydrophobic organic coatings after repeated application on both hydrophobic or wettable sands, and this could especially be an issue for an environment where periodic drought and heat are expected. Further research would be of interest to investigate the effects of tested wetting agents at higher concentrations, as well as to validate their mechanisms at microscopic/molecular level.

## Figures and Tables

**Table 1:** Summary of hydrophobic and fresh sand properties utilized in this experiment.

Sand	Bulk Density (g/cm^3^)	Particle Density (g/cm^3^)	Porosity (%)	Pore Volume (mL)	Very Coarse (1–2 mm)	Coarse (0.5–1.0 mm)	Medium (0.25–0.5 mm)	Fine (0.15–0.25 mm)	Very Fine (0.05–0.15 mm)	Organic Matter (%)	MED (M)
					Sand particle size distribution (%)						
Hydrophobic sand	1.66	2.68	38	58	6.76%	12.74%	58.73%	20.75%	1.02%	1.73%	2.2
Fresh sand	1.74	2.76	37	54	7.84%	13.50%	62.01%	16.12%	0.54%	0.27%	0.0

**Table 2:** Treatment effects on leachate volume (mL) of hydrophobic and fresh sand systems after. the first, second, and third wetting agent applications and three sequential washes following each application.

Application	Wetting Agent †	After Treatment	1st Wash ‡	2nd Wash	3rd Wash
		Volume (mL)			
Hydrophobic sand §					
1st application					
	ABP	0 b3	47 a2	56 a1	56 a1
	PoAP	1 b3	16 c2	55 a1	56 a1
	Water	17 a3	39 b2	49 b1	54 b1
2nd application					
	ABP	0 b3	45 b2	56 a1	56 a1
	PoAP	4 a3	47 a2	55 a1	56 a1
3rd application					
	ABP	4 a3	45 a2	56 a1	56 a1
	PoAP	1 b3	45 a2	55 a1	56 a1
Fresh sand					
1st application					
	ABP	17 b3	44 a2	52 b1	53 b1
	PoAP	19 a4	44 a3	52 b1	51 c2
	Water	16 c3	42 b2	54 a1	54 a1
2nd application					
	ABP	17 b3	42 c2	52 ab1	51 b1
	PoAP	16 c3	43 b2	52 b1	52 a1
	Water	21 a3	47 a2	52 a1	52 a1
3rd application					
	ABP	14 c3	44 b2	51 c1	52 b1
	PoAP	21 b4	45 a3	52 b1	52 b2
	Water	24 a3	40 c2	53 a1	53 a1

† ABP, alkyl block polymer; PoAP, polyoxyalkylene polymer. All treatment solutions were applied at 70 mL. ‡ Treated sand was washed with one pore volume of water at 58 mL for hydrophobic sand and 54 mL for fresh sand in each wash. § After the first application and three sequential washes, hydrophobic sand columns that were subjected to water only treatment became extremely hydrophobic and did not allow water to infiltrate; hence, no leachates were collected beyond the first application. Means in the same column of the same application for hydrophobic or fresh sand systems followed by the same letters were not significantly different based on Fisher’s Protected LSD at *p* < 0.05; means in the same row followed by the same numbers were not significantly different based on Fisher’s Protected LSD at *p* < 0.05.

**Table 3:** Effects of wetting agents on the dissolved and particulate organic carbon (DOC and POC, respectively) in leachates collected from fresh and hydrophobic sands after each treatment application and sequential washes.

	Fresh Sand			Hydrophobic Sand		
	1st. Application †	2nd. Application	3rd. Application	1st. Application	2nd. Application	3rd. Application
	DOC (mg)					
ABP ‡	908.6 a2 §	944.8 a1	946.4 a1	868.5 a2	923.1 a1	944.6 a1
PoAP	807.8 b3	855.7 b2	923.2 b1	720.7 b2	853.8 b1	854.7 b1
	POC (mg)					
ABP	10.5 b1	9.2 a1	8.4 b1	109.1 a1	65.2 a2	71.3 a2
PoAP	20.1 a1	9.3 a2	18.4 a1	31.6 b2	61.2 a1	52.4 b1

† The DOC and POC were calculated based on all washing events of each application. ‡ ABP, alkyl block polymer; PoAP, polyoxyalkylene polymer. Data from water treatment were excluded from this analysis as water failed to infiltrate into hydrophobic sands beyond the first application. § Means of DOC or POC in the same column of fresh or hydrophobic sand system followed by the same letters were not significantly different based on Fisher’s Protected LSD at *p* < 0.05; means of DOC or POC in the same row followed by the same numbers were not significantly different based on Fisher’s Protected LSD at *p* < 0.05.

**Table 4:** Treatment effects on total dissolved (TDOC; mg), particulate (TPOC; mg), and cumulative total organic carbon (TOC; mg) † in leachates collected from both fresh sand (FS) and hydrophobic sand (HS) columns after three treatment applications and following washing events, along with total organic carbon (OC; mg) input from the treatments.

	TDOC		TPOC		TOC		Total OC Input
	FS	HS	FS	HS	FS	HS	
ABP ‡	2799 a1 §	2736 a2	29 b2	246 a1	2828 a2	2982 a1	3864 a
PoAP	2587 b1	2429 b2	48 a2	145 b1	2634 b1	2575 b2	2997 b
Water	269 c1	70 c2	19 b1	20 c1	288 c1	90 c2	0 c

† Total dissolved and particulate organic carbon (TDOC and TPOC) was the sum of DOC and POC collected from all three applications cycles, respectively for hydrophobic or fresh sand system. Total organic carbon (TOC) was calculated by summing the TDOC and TPOC for each treatment. ‡ ABP, alkyl block polymer; PoAP, polyoxyalkylene polymer. Data from water treatment only included one application and sequential washes as water failed to infiltrate into hydrophobic sands beyond the first application. § Means in the same column of hydrophobic or fresh sand system followed by the same letters were not significantly different based on Fisher’s Protected LSD at *p* < 0.05; means in the same row followed by the same numbers were not significantly different based on Fisher’s Protected LSD at *p* < 0.05. Total organic carbon (OC) input is the combined input of three applications from both wetting agents.

## Data Availability

The data presented in this study are openly available in MOspace Institutional Repository at https://doi.org/10.32469/10355/87062.

## Supplementary Materials

The following are available online at https://www.mdpi.com/article/10.3390/nano11102577/s1. Table S1: Treatment effects on dissolved organic carbon (DOC, mg) in leachates collected from hydrophobic and fresh sand systems after the first, second, and third wetting agent applications and three sequential washes following each application. Table S2: Treatment effects on particulate organic carbon (POC, mg) in leachates collected from hydrophobic and fresh sand systems after the first, second, and third wetting agent applications and three sequential washes following each application.

## Abbreviation

ABP, alkyl block polymer; CMC, critical micelle concentration; DDW, distilled, deionized water; DOC, dissolved organic carbon; MED, molarity of ethanol droplet test; OC, organic carbon; PoAP, polyoxyalkylene polymer; POC, particulate organic carbon; SEM, scanning electronic microscopy; SOC, solid phase organic carbon; SWR, soil water repellency.
