# Supplementary material for: Certain Soil Surfactants Could Become a Source of Soil Water Repellency after Repeated Application
Source: Nanomaterials (Basel). 2021 Sep 30;11(10):2577. doi: 10.3390/nano11102577 (PMC8538948; doi:10.3390/nano11102577)
Supplement: Supplementary file 1 [file nanomaterials-11-02577-s001.zip › nanomaterials-1384138-supplementary.pdf]

Supplementary Materials

Certain Soil Surfactants Could Become a Source of Soil Water Repellency after Repeated Application

Table S1. Treatment effects on dissolved organic carbon (DOC, mg) in leachates collected from hydrophobic and fresh sand systems after the first, second, and third wetting agent applications and three sequential washes following each application.

Application	Wetting Agent†	After Treatment	1st Wash‡	2nd Wash	3rd Wash
			---------------- DOC (mg) ----------------		
Hydrophobic sand§					
1st application					
	ABP	0.2 b4¶	667.3 a1	180.4 a2	20.6 ab3
	PoAP	16.9 a4	516.3 b1	153.1 b2	34.4 a3
	Water	11.0 ab2	28.3 c1	17.9 c12	12.8 b2
2nd application					
	ABP	5.3 b3	707.3 a1	184.8 a2	26.4 a3
	PoAP	51.7 a3	616.0 b1	150.0 b2	36.1 a3
3rd application					
	ABP	60.0 a3	698.2 a1	165.5 a2	20.9 a4
	PoAP	13.5 b3	655.9 b1	149.8 a2	35.5 a3
Fresh sand					
1st application					
	ABP	229.4 a2	599.4 a1	69.2 a3	10.6 b4
	PoAP	223.6 a2	484.6 b1	74.2 a3	25.4 a4
	Water	4.7 b3	17.9 c2	36.1 b1	29.0 a1
2nd application					
	ABP	250.6 a2	619.4 a1	62.8 a3	11.9 b4
	PoAP	176.0 b2	586.3 b1	74.0 a3	19.4 b4
	Water	12.7 c2	20.3 c2	38.9 b1	33.7 a1
3rd application					
	ABP	120.9 a2	733.9 a1	71.9 b3	19.1 a4
	PoAP	84.2 b2	736.9 a1	80.4 a2	21.7 a3
	Water	10.5 c2	19.5 b1	23.1 c1	22.8 a1

† ABP, alkyl block polymer; PoAP, polyoxyalkylene polymer. All treatment solutions were applied at 70 ml.
‡ Treated sand was washed with one pore volume of water at 58 ml for hydrophobic sand and 54 ml for fresh sand in each wash.
§ After the first application and three sequential washes, hydrophobic sand columns that were subjected to water only treatment became extremely hydrophobic and did not allow water to infiltrate; hence, no leachates were collected beyond the first application.
¶ Means in the same column of the same application for hydrophobic or fresh sand systems followed by the same letters were not significantly different based on Fisher's Protected LSD at $P < 0.05$; means in the same row followed by the same numbers were not significantly different based on Fisher's Protected LSD at $P < 0.05$.

Table S2. Treatment effects on particulate organic carbon (POC, mg) in leachates collected from hydrophobic and fresh sand systems after the first, second, and third wetting agent applications and three sequential washes following each application.

Application	Wetting Agent†	After Treatment	1st Wash‡	2nd Wash	3rd Wash
			---------------- POC (mg) ----------------		
Hydrophobic sand§					
1st application					
	ABP	0.1 a3¶	89.1 a1	16.2 a12	3.8 a2
	PoAP	1.3 a3	17.2 b1	7.2 b2	5.8 a2
	Water	0.3 a2	7.0 c1	6.7 b1	6.4 a1
2nd application					
	ABP	0.0 b2	57.4 a1	5.4 b2	2.4 a2
	PoAP	13.6 a23	21.5 b1	17.3 a12	8.8 a3
3rd application					
	ABP	7.5 a2	55.4 a1	3.2 b2	5.1 b2
	PoAP	3.0 a3	8.8 b3	24.4 a1	16.4 a2
Fresh sand					
1st application					
	ABP	5.3 b1	3.2 a12	0.8 b3	1.3 a23
	PoAP	9.0 a1	1.9 a2	6.8 a1	2.4 a2
	Water	7.5 a1	2.7 a2	2.3 b2	0.6 a2
2nd application					
	ABP	3.3 a1	3.8 a1	1.0 a2	0.9 ab2
	PoAP	3.1 a1	2.0 b1	2.0 a1	2.3 a1
	Water	1.2 b1	1.3 b1	1.5 a1	0.3 b1
3rd application					
	ABP	1.7 b2	3.8 b1	1.6 a2	1.8 a2
	PoAP	3.2 a2	10.5 a1	1.1 a2	1.8 a2
	Water	1.0 b1	0.3 c1	0.3 b1	0.1 a1

† ABP, alkyl block polymer; PoAP, polyoxyalkylene polymer. All treatment solutions were applied at 70 ml.

‡ Treated sand was washed with one pore volume of water at 58 ml for hydrophobic sand and 54 ml for fresh sand in each wash.

§ After the first application and three sequential washes, hydrophobic sand columns that were subjected to water only treatment became extremely hydrophobic and did not allow water to infiltrate; hence, no leachates were collected beyond the first application.

¶ Means in the same column of the same application for hydrophobic or fresh sand systems followed by the same letters were not significantly different based on Fisher's Protected LSD at $P < 0.05$; means in the same row followed by the same numbers were not significantly different based on Fisher's Protected LSD at $P < 0.05$.
